# Chronic unexplained nausea in adults: Prevalence, impact on quality of life, and underlying organic diseases in a cohort of 5096 subjects comprehensively investigated

**DOI:** 10.1371/journal.pone.0225364

**Published:** 2019-12-19

**Authors:** Hye-Kyung Jung, Chung Hyun Tae, Chang Mo Moon, Seong-Eun Kim, Ki-Nam Shim, Sung-Ae Jung

**Affiliations:** Department of Internal Medicine, College of Medicine, Ewha Womans University, Seoul, Korea; Universidad Autonoma de Madrid, SPAIN

## Abstract

We evaluated to define the clinically significant chronic nausea in general population and to assess the prevalence of chronic unexplained nausea after exclusion of organic causes through the meticulous medical examination. Two phase studies were conducted. In phase 1, telephone survey was conducted to define the clinically significant nausea in 5000 representative subjects for a general population. Clinically significant nausea was identified by lowered quality of life if the frequency was ‘more than one day per week’. Its prevalence was 1.6% (1.4–1.8%) and about 90% of nausea was not accompanied with vomiting. In phase 2, 5096 participants in a comprehensive health-screening cohort were enrolled. We investigated demographics, gastrointestinal symptoms, somatization symptoms and health related quality of life using validated questionnaire. All participants underwent meticulous medical examinations including endoscopy, abdominal ultrasound, thyroid function test, and blood testing. Among a total of 5096 subjects (men 51.8%, mean age 47.5 ± 10.0 years), organic diseases associated with chronic nausea were reflux esophagitis, duodenal ulcer and hyperthyroidism. The prevalence of chronic unexplained nausea was 0.6% (95% CI 0.4–0.8%) and there were significant overlap with functional dyspepsia and irritable bowel syndrome. HRQoL is significantly lower in people with nausea occurring ‘more than one day per week’ in a general population. Most chronic nausea was not accompanied with vomiting. Chronic unexplained nausea is uncommon affecting only 0.6% of the population but are more likely to report functional dyspepsia and irritable bowel syndrome.

## Introduction

Nausea is the subjective, unpleasant sensation of the need to vomit, and while it may be accompanied by vomiting although many people experience prolonged nausea without ever having to vomit [1]. Nausea is a complex subjective symptom that ranges from uncomfortable to agonizing and may accompany many diseases and conditions including diseases within and outside the gut as well as by circulating toxins, drugs and metabolic disorders [2–3]. Visceral obstruction and inflammation of gut and solid organ may cause acute nausea and gut sensorimotor dysfunction often elicit chronic nausea. Chronic nausea with or without vomiting associated with delayed gastric emptying is defined as gastroparesis, however most subjects complaining of chronic nausea have normal gastric emptying [4].

Chronic unexplained nausea and vomiting disorders are included as part of the classification of functional gastrointestinal disorders (FGIDs). In the Rome III criteria, nausea and vomiting disorders consisted of 4 subcategories, namely chronic idiopathic nausea, functional vomiting, cyclic vomiting syndrome and rumination syndrome in adults [5], while the Rome IV criteria collapsed functional nausea and vomiting disorders (FNVD) into three subcategories as follows; chronic nausea and vomiting syndrome (CNVS), cyclic vomiting syndrome (CVS), and cannabinoid hyperemesis syndrome [1]. Rome IV criteria changed these criteria because of the poor evidence demonstrating a differentiation between nausea and vomiting disorders in terms of pathogenesis or treatment [6]. However, in our referral clinics, we often encounter patients complaining of only nausea without accompanying vomiting that can be very challenging to diagnose and treat. The lack of understanding about chronic unexplained nausea adds distress for both patients and physician, and may exacerbate comorbid disability or psychological distress in these patients.

We aimed to 1) define the prevalence of clinically significant nausea in a general population, and 2) to further evaluate the potential underlying explanations of uninvestigated nausea and estimate the prevalence of chronic unexplained nausea and its comorbidity and impact on quality of life in the community.

## Methods

### Study population

**Fig 1:**
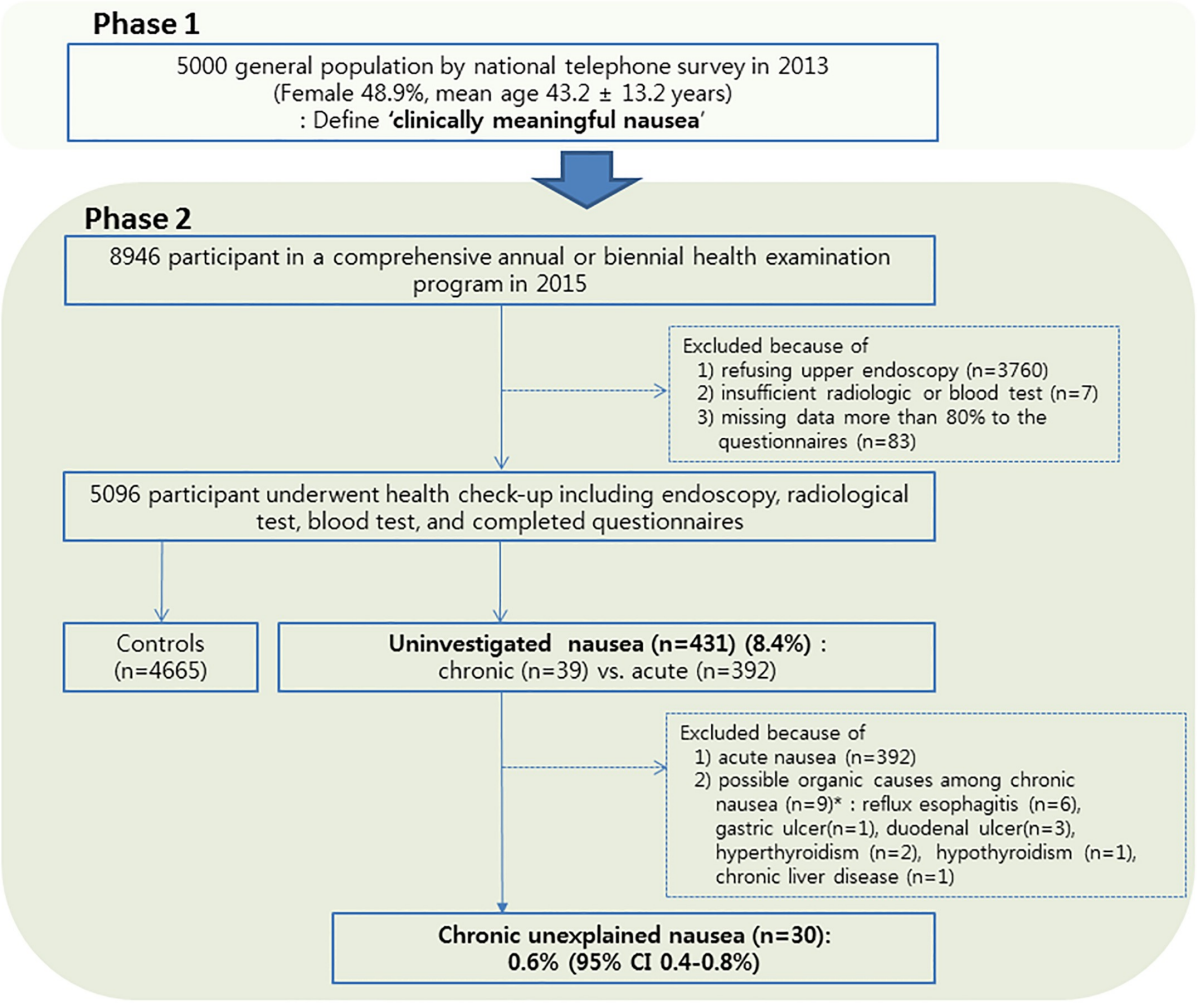
Study flow. Phase 1 is conducted in 5000 general population. Phase 2 is conducted in 5096 subjects who participated in medical health check-up;*data are not mutually exclusive.

There were two phases of the present study. In phase 1, a population-based, cross-sectional study was conducted by computer aided telephone interview (CATI) in 5000 Koreans between the ages of 20–60 years (Fig 1). This phase 1 study was conducted to find a definition of clinically significant nausea as a sub-study of previously reported epidemiological studies [7]. Based on the population structure of the 2010 Census of Population and Housing for adults aged between 20 and 69, the targets of CATI were allocated proportionally according to gender, age, and regional population composition in Seoul, six metropolitan cities and eight provinces in Korea [8]. Target population was randomly selected from the list of computer populations, and the data collection status by date and interviewee was verified in real time using CATI to structure the verification procedure. During the study, a total of 74,730 calls were made and 44,891 fixed-line calls were successfully connected. The overall access rate is 60.1%. Of the 44,891 cases, 5,000 people gave full interviews, generating an overall response rate of 11.1%.

The phase 2 study was conducted among men and women aged 19 years or older who underwent a comprehensive annual or biennial health examination at the clinics of the Ewha Womans University Health Promotion Center in Seoul, South Korea in 2015. In Korea, the Industrial Safety and Health Law stipulates that health check-ups are provided free of charge for all employees every year or every two years. More than 70% of participants were employees of several types of companies and their spouses and 30% paid by themselves. The present study included all study participants who received a comprehensive health check-up. A total of 8946 consecutive subjects were included in the present study. Since stomach cancer is a common country in Korea, participants can choose the upper gastrointestinal (GI) contrast examination or gastroscopy according to their intention for stomach cancer screening. Among them, subjects who received the upper GI contrast examination, not the upper endoscopy were excluded (n = 3,760) (Fig 1). We further excluded the subjects without sufficient examination including blood and radiologic tests (n = 7), as well as participants who had missing data on more than 80% to the questionnaire conducted during the examination (n = 83). Finally, 5096 subjects were enrolled in the study. (Fig 1).

The study protocol was approved by the Institutional Review Board of Ewha Womans University (EUMC-2018-06-011-001). The requirement for informed consent was waived because we were using unidentified data collected routinely during the health screening process.

### Questionnaires for health-related quality of life (HRQoL), GI symptoms, and somatization scores

In phase 1, the survey questions were about the presence of nausea and HRQoL. Chronic nausea was defined when bothersome nausea is present for the last 3 months with symptom onset at least 6 months prior to diagnosis. The HRQoL was assessed using the EuroQoL (EQ-5D) [9]. The EQ-5D was developed with the intent of constructing a simple, self-administered instrument. The descriptive system records the level of self-reported problems on each of the five dimensions of the classification (mobility, self-care, usual activities, pain/discomfort, anxiety/depression). The EQ-5D index was calculated using a previously documented method [10, 11]. In order to define a clinically significant nausea, the difference in HRQoL was analyzed according to the frequency of nausea.

In phase 2, all participants completed GI symptom questionnaires including the validated Korean Rome III questionnaire (Rome III-K) for diagnosis of FGIDs, the Somatic Symptom Checklist (SSC) and EQ-5D. The Rome III-K contains 25 GI symptoms and 17 items including past illness, socio-demographic variables, and a valid measure of non-GI somatic complaints [12–13]. Rome III-K has shown adequate reliability and validity in an outpatients setting. The Rome III-K is reliable with a median Cronbach’s α value of 0.83 (range, 0.71–0.97) and satisfactory construct validity.

Uninvestigated nausea was defined as the same symptoms criteria with and without underlying organic causes. Among them, subjects who had nausea that did not fulfill the definition of symptom duration of chronic uninvestigated nausea were classified as an acute nausea group (S1 Data).

Chronic unexplained nausea was defined as follows; bothersome nausea usually not accompanied with vomiting and no abnormalities at upper endoscopy or metabolic disease that explains the nausea [14].

Irritable bowel syndrome (IBS), IBS subtypes and functional dyspepsia (FD) and its subtypes of postprandial distress syndrome (PDS) and epigastric pain syndrome (EPS) were defined by Rome III criteria [14, 15].

The SSC was used for evaluate 17 non-GI symptoms or illnesses, and respondents were instructed to indicate the frequency of each symptoms (on a scale of 0, indicating not a problem, to 4, indicating occurs daily) and the bothersome of each symptom on a scale of 0, not a problem, to 4, extremely bothersome when it occurs during the past [13].

### Socio-demographic and clinical data collection

In phase 2, comprehensive health examinations were conducted at the clinics of the Ewha Womans University Hospital Health Promotion Center in Seoul. Demographic characteristics, medical and social history, GI symptoms were collected by self-administered standardized questionnaires. Alcohol drinking was defined as more than 61 g/week for males and 41 g/week for females [16]. Hypertension was defined as a self-reported history of hypertension, current use of anti-hypertensive drugs, or a systolic blood pressure of at least 140 mmHg or a diastolic blood pressure of at least 90 mmHg. Diabetes mellitus was defined as a fasting blood sugar of at least 126 mg/dL, a self-reported history of diabetes mellitus or current use of hypoglycemic agents. Uncontrolled diabetes mellitus was defined as cases where HbA1c was 7.0% or more. Chronic liver disease was defined as chronic viral hepatitis, alcoholic liver disease or liver cirrhosis established by ultrasonography and laboratory findings. Renal insufficiency was defined as chronic kidney disease stage 3 or more.

Serum total cholesterol, triglyceride, other cholesterol profiles and glucose were measured using colorimetric methods by an autonomic analytic instrument (Hitachi 7600–110 Automatic analyzer, Hitachi, Tokyo, Japan) in blood samples collected after at least 12 h of fasting. Thyroid function test, C-reactive protein, and complete blood cell counts were also measured at the Department of Laboratory Medicine which has been accredited by the Korean Society of Laboratory Medicine and the Korean Association of Quality Assurance for Clinical Laboratories, and participates in the College of American Pathologists Survey Proficiency Testing.

Upper endoscopy was performed by three experienced certified gastroenterologists using a videogastroscope (GIF-H290 EVIS LUCERA ELITE, Olympus, Tokyo, Japan). Abdomen ultrasonography (Philips EPIQ 7G, Philips, N.Y, U.S.A) was performed to evaluate the abnormalities of hepatobiliary system and kidney. Abdomen ultrasonography was performed by two radiologists with more than 10 years of clinical experience. Routine urinalysis and chest PA were also obtained.

### Analysis

We defined clinically significant nausea as a statistically significant decline in the EQ- 5D_index_ as the frequency of occurrence. We compared demographic data of those with chronic nausea and those of all other patients using a Chi square test for categorical variables, and an independent sample t-test for continuous data with mean and standard deviation (SD). EQ-5D index was described by their mean with SD. We converted data from SSC into four-percentiles and evaluated whether the severity of the somatization tends to increase in the groups with chronic unexplained nausea through the results of linear by linear association. To evaluate the predictors of chronic unexplained nausea, we estimated odds ratios (ORs) with 95% confidence intervals (CIs) using multiple logistic regression analysis.

We conducted the sensitivity analysis to test the robustness of our results. Firstly, it was tended to verify the hypothesis that the definition of “clinically meaningfulness” is defined as a group with 25% low QOL instead of below 10% of QOL. Secondly, the criterion of nausea more than once a week corresponding to the Rome IV Criterion is less than 10% of QOL, but is not used as the criterion in primary analysis because there is no statistical difference from the QOL of patients with less than once a week. However, sensitivity analysis was performed with Rome IV criteria to compare the results of previous studies.

All statistical analyses were performed using SPSS for Window version 21.0 (SPSS Inc., Chicago, IL, USA). All statistical analysis was 2-sided, and P < 0.05 was considered statistically significant.

## Results

### Phase 1: Defining the prevalence of clinically significant chronic nausea in a general population

In phase 1 study, 5000 subjects completed the interview (females 48.9%, mean age 43.2 ± 13.2 years). The demographic composition of this data was similar to that of the national statistics undertaken by the Ministry of Security and Public Administration in August 2012 [17]. In EQ-5D index distribution, the histogram plot results of EQ5D show negative skewness (mean 0.93, median 0.95, mode 0.95) and the 0.87 score corresponding to the lower 10 percentile of EQ-5D was considered as cutoff point. The group with a poor HRQoL, defined as mean HRQoL lower than 0.87, was the subjects with nausea ‘one day a week’, ‘more than one day a week’ and ‘daily’ symptom group. The OR for the poor HRQoL increased as the frequency of nausea increased (Table 1).

**Table 1:** Health-related quality of life according to the frequency of nausea in general population; Phase 1.

Frequency of nausea	No.	*Odd ratios ± 95% CI	EQ-5D_index_ Mean ± SD	**Subset for alpha = 0.05			
				1	2	3	4
Every day	15	7.36 (2.65–20.80)	0.814 ± 0.124	0.814			
More than one day a week	66	5.90 (3.51–9.92)	0.817 ± 0.195	0.817			
One day a week	72	2.90 (1.62–5.18)	0.868 ± 0.124		0.868		
Two to three days a month	237	2.44 (1.72–3.46)	0.881 ± 0.098		0.881	0.881	
One day a month	212	2.33 (1.61–3.38)	0.885 ± 0.105		0.885	0.885	
Less than one day a month	303	1.44 (0.99–2.08)	0.907 ± 0.083			0.907	0.907
Never	4095	1.00	0.925 ± 0.064				0.925

* After setting the poor HRQoL group according to the frequency of nausea with EQ-5D 0.87 corresponding to the lower 10 percentile as cut off, odd ratios of poor HRQoL was calculated;

**Post-hoc analysis was performed to find the mean difference of EQ-5D_index_ according to the frequency of nausea; CI, confidence interval.

Post-hoc analysis was conducted to see the statistical mean differences in the HRQoL according to the frequency of nausea, and we finally confirmed ‘the clinically significant nausea’ as having nausea ‘more than one day a week’ (Table 1). With HRQoL’s cutoff, vomiting ‘more than one day a week’ showed poor HRQoL. Clinically significant nausea was 1.6% (1.4–1.8%) (81/5000) and vomiting was 0.2% (0.1–0.3%) (12/5000). Only 11.1% (9/81) of those with nausea had vomiting, and about 90% had only nausea symptoms.

Among 5000 respondents, the prevalence of uninvestigated dyspepsia by Rome III criteria was 7.7% (95% CI 7.0–8.4%). The prevalence of EPS and PDS were 4.2% and 5.6%, respectively. The prevalence of symptomatic gastroesophageal reflux disease was 7.1% (95% CI 6.4–7.8%) and that of IBS was 3.5% [8].

### Phase 2: Underlying causes of uninvestigated nausea and epidemiologic features of chronic unexplained nausea in the community

Among initial 8946 participants, a total of 5096 subjects was included; the mean age was 47.5 ± 10.0 years and 51.8% were male. The mean BMI was 23.6 ± 3.1 kg/m^2^ (range 11.0–42.0). The basic characteristics of initial included subjects were compared with excluded subjects (n = 3850) as follows; the mean age of the inclusion group was higher than that of the exclusion group with male predominance (44.1 ± 9.1 years, p < 0.01; 45.3%, p < 0.01). Hypertension was more common in the inclusion group than in the exclusion group (10.6% vs. 8.4%, p < 0.01), however, BMI (kg/m^2^) (23.6 ± 3.2 vs. 23.7 ± 3.5, p = 0.45), smoking (18.6% vs. 19.7%, p = 0.18) and DM (3.6% vs. 2.9%, p = 0.20) were not different between the two. Therefore, excluding these participants might not significantly affect the results of this study. Among inclusion group, controls were defined as subjects without nausea (Fig 1).

The prevalence of uninvestigated nausea was 8.4% (431/5096), and among them, acute uninvestigated nausea was detected in 7.7% (392/5096) and chronic uninvestigated nausea 0.8% (39/5096) (Fig 1). The participants with chronic uninvestigated nausea were more likely to be younger, current smoker, alcohol drinkers, and having asthma. However, there was no difference of medical history of hypertension, diabetes mellitus and obesity according to the presence of chronic nausea compared with controls (Table 2). Serum r-GT level was significantly higher in participants with uninvestigated nausea compared to controls. However, there were no differences of other laboratory findings including hemoglobin, lipid profiles, erythrocyte sedimentation rate, and protein and so on.

**Table 2:** Baseline characteristics of uninvestigated nausea in health check-up group; Phase 2 study.

	Controls (n = 4665)	Acute uninvestigated nausea (n = 392)	Chronic uninvestigated nausea (n = 39)	P value
**Age**	47.7 ± 9.9	45.6 ± 10.4	41.7 ± 10.0	<0.01
** < 40 years (%)**	911 (19.5)	111 (28.3)	15 (38.5)	<0.01
**Female sex (%)**	2227 (47.7)	208 (53.1)	19 (48.7)	0.12
**BMI (kg/m**^**2**^**)**	23.7 ± 3.2	23.2 ± 3.3	23.0 ± 3.2	0.02
**Obesity (≥ 25 kg/m**^**2**^ **) (%)**	1737 (37.3)	135 (34.4)	11 (28.2)	0.28
**Hb (g/dL)**	14.3 ± 1.6	14.2 ± 1.6	14.7 ± 1.5	0.20
**Albumin (g/dL)**	4.1 ± 0.3	4.1 ± 0.2	4.1 ± 0.3	0.29
**Total cholesterol (mg/dL)**	197.6 ± 34.2	199.3 ± 36.9	196.3 ± 36.5	0.62
**HDL-cholesterol (mg/dL)**	55.7 ± 13.2	57.0 ± 14.8	58.7 ± 12.6	0.06
**Triglyceride (mg/dL)**	118.6 ± 80.3	123.3 ± 108.8	115.9 ± 78.6	0.55
**r-GT (mg/dL)**	34.1 ± 38.7	38.7 ± 52.3	53.3 ± 59.7	0.01
^†^**ESR (mm/h)**	10.3 ± 8.1	10.4 ± 8.4	10.8 ± 10.4	0.91
**HbA1c (%)**	5.6 ± 0.7	5.5 ± 0.7	5.4 ± 0.5	0.14
**Alcohol (n = 4986) (%)**	1005 (22.0)	125 (32.8)	19 (48.7)	<0.01
**Current smoker (n = 4884)(%)**	804 (18.0)	89 (23.6)	15 (39.5)	<0.01
**Diabetes mellitus (%)**	253 (5.4)	14 (3.6)	1 (2.6)	0.21
**Hypertension (%)**	609 (13.1)	49 (12.5)	4(10.3)	0.83
**Asthma (%)**	206 (4.4)	53 (7.7)	7 (17.9)	<0.01
**CVA (%)**	29 (0.6)	0 (0)	1(2.6)	0.08
**Coronary artery disease (%)**	43 (0.9)	8(2.0)	1 (2.6)	0.06
**Somatization (%)**	151 (3.2)	40 (10.2)	9 (23.1)	<0.01

^†^ ESR, erythrocyte sedimentation rate.

### Organic explanations for chronic uninvestigated nausea

The causes of chronic uninvestigated nausea are diverse and related to the GI tract and non-GI causes [3]. Possible organic causes were found more frequently in the group with chronic uninvestigated nausea compared to the subjects with acute nausea and controls (23.1% vs. 19.4% vs. 14.0%, P < 0.01) (Table 3). Compared with the control group, reflux esophagitis and duodenal ulcer were observed significantly higher in the group with chronic uninvestigated nausea (15.4% vs. 6.2%, P = 0.01; 7.7% vs. 1.5%, P = 0.01, respectively), however, there was no significant difference in gastric ulcer disease, esophageal or stomach cancer, chronic viral hepatitis, liver cirrhosis, hepato-biliary stones, or gall bladder wall thickening/adenomyomatosis. In addition, hyperthyroidism was more frequently detected in chronic uninvestigated nausea group compared to controls (5.1% vs. 1.0%, P = 0.03). However, other non-GI diseases such as uncontrolled diabetes mellitus, chronic renal insufficiency, kidney stone, bradycardia, and hypothyroidism were not significantly different between subjects with chronic uninvestigated nausea and controls.

**Table 3:** Possible organic causes of uninvestigated nausea.

Number (%)	Controls (n = 4665)	Acute uninvestigated nausea (n = 392)	Chronic uninvestigated nausea (n = 39)	P value
**Organic disease**	655 (14.0)	76 (19.4)	9 (23.1)	< 0.01
**Reflux esophagitis**	288 (6.2)	33 (8.4)	6 (15.4)	0.01
**Esophageal cancer**	1 (<0.1)	0 (0)	0 (0)	0.95
**Gastric ulcer**	146 (3.1)	11 (2.8)	1 (2.6)	0.92
**Duodenal ulcer**	71 (1.5)	2 (0.5)	3 (7.7)	0.01
**Stomach cancer**	7 (0.2)	1 (0.3)	0 (<0.1)	0.85
**Chronic liver disease**	175 (3.8)	9 (2.3)	1 (2.6)	0.31
**Uncontrolled DM (n = 4272)**	184 (4.7)	15 (4.5)	0 (0)	0.44
**GB stone**	50 (1.1)	5 (1.3)	0 (0)	0.75
**GB wall thickening**	18 (0.4)	1 (0.3)	0 (0)	0.85
**Renal insufficiency**	1 (<0.1)	0 (0)	0 (0)	0.95
**Renal stone**	101 (2.2)	12 (3.1)	0 (0)	0.32
**Bradycardia**	3 (0.1)	0 (0)	0 (0)	0.87
**Gastrectomy**	10 (0.2)	2 (0.5)	0 (0)	0.48
**Cholecystectomy**	29 (0.6)	5 (1.3)	0 (0)	0.27
**Hyperthyroidism**	46 (1.0)	5 (1.3)	2 (5.1)	0.03
**Hypothyroidism**	42 (0.9)	1(0.3)	1(2.6)	0.21

All Data are not mutually exclusive; Gastric and duodenal ulcer was included as having active or healing stage of ulcer; Uncontrolled DM, HbA1c >7; Renal insufficiency, CRF stage 3 or more.

### Prevalence of chronic unexplained nausea and overlap with FGIDs and somatization

**Fig 2:**
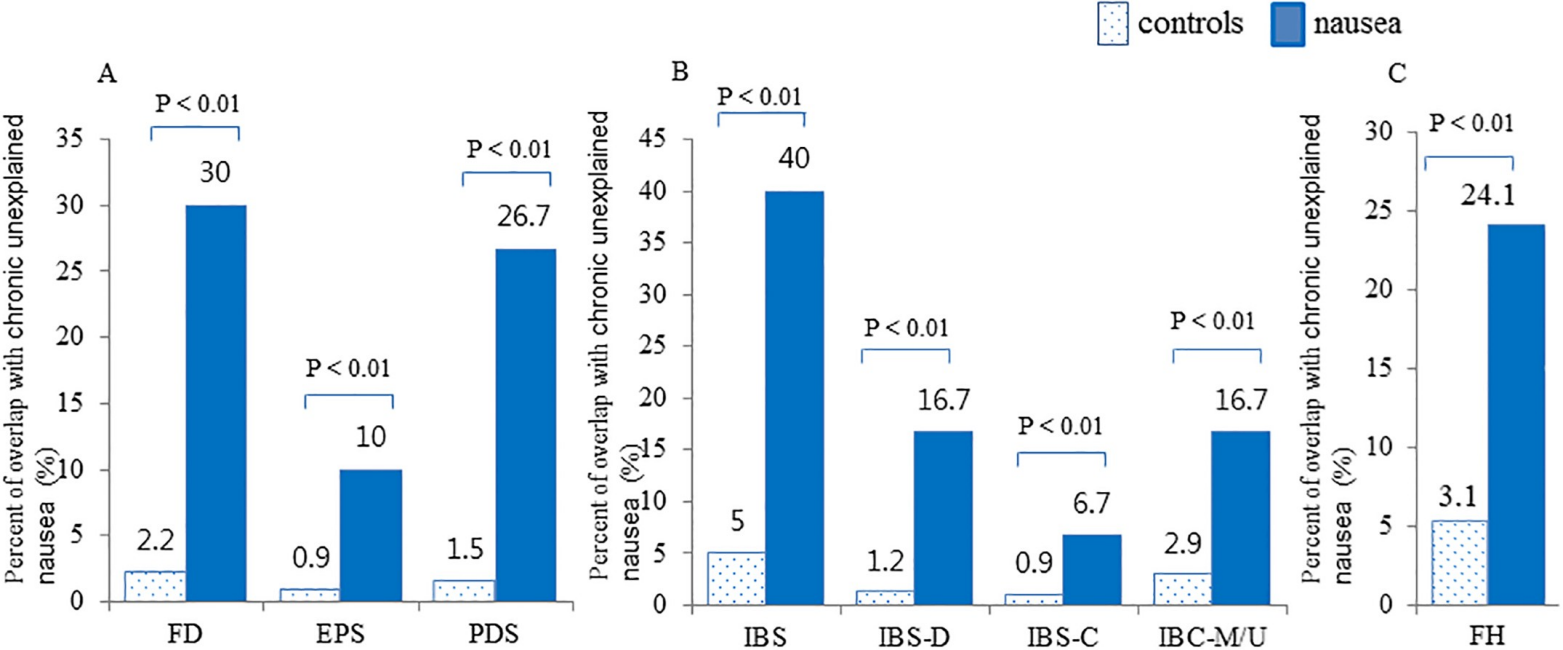
Overlap of chronic unexplained nausea with functional gastrointestinal disorders. The proportion of functional gastrointestinal disorders is significantly higher in nausea group compared to controls. FD, functional dyspepsia; EPS, epigastric pain syndrome; PDS, postprandial distress syndrome; IBS, irritable bowel syndrome; IBS-D, diarrhea predominant IBS; IBS-C, constipation predominant IBS; IBS-M/U, mixed or unspecified IBS; FH, functional heartburn.

Of the total 5096 people, the prevalence of chronic unexplained nausea was 0.6% (95% CI 0.4–0.8%) after excluding the subjects who had any organic cause related to nausea. The prevalence of FD was 3.2% (95% CI 2.7–3.7%) and that of IBS was 6.8% (95% CI 6.1–7.5%). Among subjects with chronic unexplained nausea, the proportion of FD was significantly higher than that in controls (30.0% vs. 2.2%, P < 0.01) and both PDS and EPS were more frequently detected, suggesting nausea is a feature of FD (Fig 2). The proportion with IBS was also more frequently detected in chronic unexplained nausea compared to controls (40.0% vs. 5.0%, P < 0.01) and all subtypes more frequently overlapped in chronic unexplained nausea, compared to controls, again suggesting this symptom is a feature of IBS. The proportion of subjects who had weekly bothersome heartburn and/or acid regurgitation without endoscopic organic disorders was significantly higher in subjects with chronic unexplained nausea than that in controls (24.1% vs. 3.1%, P < 0.01). We converted data from SSC into four-percentiles and the highest fourth-percentile of SSCs was 43.3% in subjects with chronic unexplained nausea, compared with 8.9% of the control group (P < 0.01). As a result of the linear by linear association, the proportion of chronic unexplained nausea was significantly correlated with severity of the somatization (P < 0.001).

### Quality of life of chronic unexplained nausea

The EQ-5D_index_ of chronic unexplained nausea was significantly lower compared with controls (0.89 ± 0.08 vs. 0.93 ± 0.04, P < 0.01) (Table 4). Similarly, the EQ-5D measured by visual analogue scale was significantly lower in subjects with chronic unexplained nausea compared to controls (62.2 ± 13.9 vs. 77.1 ± 13.8, P < 0.01). According the domains of the EQ-5D, the domain of anxiety / depression, pain / discomfort, usual activity and mobility were significantly lower in chronic unexplained nausea compared to controls, while self-care was not limited. The EQ-5D_index_ of chronic unexplained nausea was lower than in IBS (0.91 ± 0.07, P < 0.01), FD (0.90 ± 0.10, P < 0.01), diabetes mellitus (0.92 ± 0.06, P < 0.01), hypertension (0.92 ± 0.07, P < 0.01), asthma (0.92 ± 0.07, P < 0.01), and higher than in CVA (0.87 ± 0.10, P < 0.01).

**Table 4:** EQ-5D_index_ for overall health status and frequency of any impairment in health-related quality of life in chronic unexplained nausea.

	Controls (n = 4665)	Chronic unexplained nausea (n = 30)	P value
**EQ-5D**_**index**_	0.93 ± 0.04	0.89 ± 0.08	<0.01
**EQ-5Dvas**	77.1 ± 13.8	62.2 ± 13.9	<0.01
**Anxiety/depression**			
**1**	3649 (78.2)	15 (50.0)	<0.01
**2**	992 (21.3)	12 (40.0)	
**3**	24 (0.5)	3 (10.0)	
**Pain/discomfort**			
**1**	3618 (77.6)	9 (30.0)	<0.01
**2**	1006 (21.6)	21 (70.0)	
**3**	41 (0.9)	0 (0)	
**Usual activity**			
**1**	4548 (97.5)	24 (80.0)	<0.01
**2**	116 (2.5)	6 (20.0)	
**3**	0 (0)	0 (0)	
**Mobility**			
**1**	4447 (95.3)	25 (83.3)	<0.01
**2**	217 (4.7)	5(16.7)	
**3**	1 (< 0.1)	0 (0)	
**Self-care**			
**1**	4630 (99.2)	29 (0.6)	0.20
**2**	31 (0.7)	1 (3.3)	
**3**	4 (0.1)	0 (0)	

### Multivariate analysis for predictors for chronic unexplained nausea

As shown Table 5, stepwise logistic regression analysis was performed to select the significant independent predictors for having chronic unexplained nausea. In model 1, chronic unexplained nausea was found to be associated with younger age less than 40 years (OR 2.49, 95% CI 1.16–5.24, P = 0.02), female gender (OR 3.09, 95% CI 1.24–7.69, P = 0.02), alcohol drinking (OR 2.85, 95% CI 1.23–6.60, P = 0.01), and current smokers (OR 3.88, 95% CI 1.53–9.76, P < 0.01). After adjusting by age, sex, alcohol drinking and current smoker in model 2, asthma (OR 2.83, 95% CI 1.04–7.68, P = 0.04) and somatization (OR 5.44, 95% CI 2.44–12.20, P < 0.01) were significantly associated with chronic unexplained nausea.

**Table 5:** Multivariate analysis for predictors of chronic unexplained nausea.

N (%)	Univariate analysis			Multivariate analysis: model 1			Multivariate analysis: model 2		
	OR	95% CI	*p* value	OR	95% CI	*p* value	OR	95% CI	*p* value
**Young age** **<40 years**	3.15	1.52–6.50	0.01	2.49	1.16–5.24	0.02	2.56	1.19–5.51	0.02
**Female gender**	1.25	0.61–2.60	0.54	3.09	1.24–7.69	0.02	2.20	0.87–5.58	0.09
**Current smoking**	3.21	1.53–6.76	0.01	3.88	1.53–9.76	<0.01	2.92	1.17–7.32	0.02
**Chronic alcohol use**	2.71	1.31–5.60	0.02	2.85	1.23–6.60	0.01	2.44	1.05–5.66	0.04
**Somatization**	8.31	4.00–17.24	<0.01				5.44	2.44–12.20	<0.01
**Asthma**	5.41	2.18–13.38	<0.01				2.83	1.04–7.68	0.04

OR, odd ratio; CI, confidence interval; Alcohol use was defined as more than 61 g/week for males and 41 g/week for females

### Sensitivity analysis

In sensitivity analysis, the prevalence of nausea was changed according to its’ definition. In phase 1, participants having nausea more than once per month had a low QOL of less than 25%, based on these criteria the prevalence of nausea was 12% (95% CI 11.0%– 13.0%). The prevalence of clinically significant nausea at least one day per week that met the Rome IV criteria was 3.0% (95% CI 2.5–3.5%) and that of vomiting 0.4% (0.2%– 0.5%). However, the former definition was judged not to be clinically relevant, therefore, a detailed statistical analysis of phase 2 was conducted using the definitions of Rome IV criteria.

In phase 2, the prevalence of uninvestigated nausea was 9.1% (464/5096) and there was no difference from the previous analysis, except that women were at a higher rate of acute nausea by the definition of at least one day per week, as compared to more than one day per week (S1 Table). In model by Rome IV criteria, RE was remained as statistically significant organic cause for chronic uninvestigated nausea, however, duodenal ulcer and hyperthyroidism were similar in direction to those reported for chronic nausea, but they were not statistically significant, due to small sample size (S2 Table). The prevalence of chronic unexplained nausea by Rome IV criteria was 1.45% (95% CI 1.12–1.78%) after excluding the subjects who had any organic cause related to nausea. The predictors for chronic unexplained nausea by Rome IV criteria were also similar in direction and magnitude except current smoking did not reach the statistical power (S3 Table).

## Discussion

There were limited epidemiologic studies of chronic nausea. In Rome IV criteria, nausea and vomiting were considered as the similar disease entity by unifying them into the CNVS [1]. However, in the present study, about 90% of subject with nausea are not accompanied by vomiting, but only nausea. In this study, compared to controls, all cases with nausea had a lower HRQoL, and the mean HRQoL for the nausea group corresponding to the Roman IV criterion was less than 10% of EQ-5D, but their HRQoL was not statistically different from the group having ‘2–3 days a month’ of nausea. Therefore, we defined the clinically significant nausea as having nausea ‘more than one day a week’.

It is important to introduce a dynamic threshold concept to understand the pathological conditions underlying nausea. This population based epidemiological study in phase 1 has determined the bothersome nausea as having symptoms ‘more than one day a week’ by defined as statistically significant frequency of nausea that degrades HRQoL. Clinically significant nausea was 1.6% (1.4–1.8%) and vomiting was 0.2% (0.1–0.3%) and about 90% of nausea was not accompanied with vomiting. In phase 2, we identified the subjects with uninvestigated nausea with the definition of nausea in phase 1 and excluded the subjects with organic nausea through comprehensive medical examination. Finally, this study confirmed that the prevalence of chronic unexplained nausea was 0.6% (95% CI 0.4–0.8%) in large health check-up cohort. In sensitivity analysis with the Rome IV criteria, the prevalence of chronic unexplained nausea was assumed as 1.45% in phase 2 cohort, similar or slightly lower than previous study studies [18].

Recently, Aziz et al. [18] reported that FNVD by Rome IV criteria was detected in 2.2% of general population and the prevalence of CNVS, defined as having nausea occurring ‘at least 1 day per week’ and/or one or more vomiting per week, ranged from 0.8% to 1.2%. In that population, CVS was predominant among CNVS and other main subset of CNVS was cannabinoid hyperemesis syndrome, especially in United States. Because the use of cannabis or medical marijuana is illegal in Korea, cannabinoid hyperemesis syndrome in Korea is extremely rare [19], therefore, our prevalence was lower than the study conducted in the United States, England and Canada.

Another novel finding is that a comprehensive examination was conducted to investigate various organic causes of nausea. Nausea is accompanied with various GI or non-GI diseases in clinical practice and is not readily identifiable organic abnormalities. In the present study, reflux esophagitis, duodenal ulcer and hyperthyroidism provoked nausea. Most frequent combined GI disease is reflux esophagitis. Nausea was significantly prevalent compared to controls as 15.4% of subjects with reflux esophagitis. In the previous long-term follow-up study of reflux esophagitis, 10% patients with reflux esophagitis reported nausea and this group showed poor response to anti-secretory treatment with milder disease severity, compared to the reflux esophagitis patient group not complaining nausea [11]. And active duodenal ulcer, not gastric ulcer, was frequently accompanied with chronic nausea. The pathogenesis of development of nausea in subjects with duodenal ulcer is unknown. However, it is likely that the gastric emptying was delayed due to gastric outlet passage disturbance in duodenal ulcer. Among thyrotoxicosis patients, 36% of cases reported abdominal symptoms as chief complaints and nausea was reported in 28% [20]. In hyperthyroidism, tachygastria and delayed gastric emptying was frequently displaced [21]. In sensitivity analysis with Rome IV criteria, duodenal ulcer and hyperthyroidism were similar in direction to those reported for chronic nausea, but they were not statistically significant. Due to the low prevalence of chronic nausea, it may have caused type I/ II errors by not reaching the appropriate number of samples. Further study of the larger sample size is required to prove this.

Experiencing chronic unexplained nausea at least ‘more than one day per week’ brings significantly lower HRQoL, especially pain/discomfort domain. The HRQoL in subjects with chronic unexplained nausea was lower than those of FD and IBS, and, was as low as CVA. Chronic unexplained nausea, recognized by nausea itself, is degraded quality of life, but can be further impacted by the accompanying comorbidities. The chronic unexplained nausea increased the OR to be accompanied with high somatization tendency by five times and significantly more frequently accompanied with FGIDs, such as FD/IBS, than with controls. Patients with somatization symptom experience alterations of attention, anticipation and pain memories [2]. In IBS patients, high somatization was related to the increased insular activation that is correlated with heightened sensory coding of stimuli [22]. Therefore, manifestation of chronic nausea might be related with brain-gut interaction in heightened somatic awareness.

Other independent predictors for chronic unexplained nausea were younger age, smoking, alcohol drinking and asthma. In a study on the smoking, preloading nicotine on quitting smoking decreased cigarette consumption, but significantly increased nausea [23]. Binge drinking was associated with GI symptoms among women with IBS, the studies of role of alcohol in FGIDs have had inconsistent findings [24]. Interestingly, in subjects with asthma, chronic unexplained nausea often accompanied 3 times as much as controls. Systematic review showed that long-term corticosteroid exposure in asthma associated with GI adverse effects, such as nausea and vomiting [25]. Besides, nausea is frequently reported as common drug adverse effects in subjects with asthma using long time phosphodiesterase 4 inhibitors [26]. Another possible explanation is the association between gastro-esophageal reflux (GER) and nausea. GER has been liked to chronic cough, hoarseness or asthma. GER/GERD is frequently detected in patients with asthma in 34–89% [27]. GER can provoke cough directly by micro-aspiration, or trigger or aggravate the respiratory symptoms by vagal nerve stimulation [28]. In GER/GERD subjects, nausea was frequently accompanied; however, any causality is not clear. IBS, FD and multiple FGIDs share an association with atopic conditions, including asthma [28]. The mechanism of this link may be co-expression of a genetic/environmental link related to microscopic inflammation. Imbalance of the stress axis response in which neurogenic inflammation with a cytokine TH2 cytokine bias leads to exacerbation of atopic disease might be related with expression of FGIDs [29].

The available treatment options remain unsatisfactory because it is limited identified pathophysiological mechanisms. Factor analysis revealed that the symptom of nausea and vomiting is a distinct group different from PDS group consisting of fullness, early satiation and bloating and epigastric pain group [30]. Recent studies showed a correlation between the gastric emptying or hypersensitivity and nausea [30, 31], and the role of complex electrical slow wave abnormalities and loss of interstitial cells of Cajal as a pathophysiological mechanism underlying patients with chronic unexplained nausea and vomiting as well as in gastroparesis were suggested [32].

The strength of the present study is that we conducted the verification about the clinically significant nausea based on HRQoL in general population in phase 1. We invited large target populations of 5000 people who were stratified by region, socioeconomic conditions, age and gender across the country with a well-designed highly effort telephone survey method. Second, in phase 2, we assessed the high quality data through meticulous examination, such as upper endoscopy, abdomen ultrasonography, thyroid function test and other blood test. The third advantage is that the large size of study population in phase 2 and 56.9% of participants in the overall medical check-up program were included, which can allow the multi-variate analysis even though low prevalence of chronic unexplained nausea.

However, there are limitations to this research. First, in phase 2, it is possible that the study subjects were voluntarily involved in a medical check-up and that the volunteer bias might be involved. Two thirds of those who have participated a medical check-up are those who must be carried out according to the Industrial Safety and Health Law Stipulates, therefore, they performed the medical check-up without any charge, it might be less biased compared to voluntary participants. Those who voluntarily participate may be groups that are more biased from the general population. However, meticulous clinical examination including endoscopy and abdomen ultrasonography may not be realistic in the general population. Therefore, it is likely that our screening cohort is the best alternative to maintain the possible generalizability. Second, self-reported questionnaires were used to evaluate the GI and comorbidities. A structured clinical interview is known as the gold standard and is superior to self-report measure for psychiatric comorbidity [30], even though questionnaires for GI and somatization have been validated. Third, these two phase studies were conducted as cross-sectional studies, so it is difficult to know causality. For example, asthma is presented as a predictor for nausea, but at the same time, nausea might provoke or aggravate the asthma. Fourth, it is possible that non-responder bias were involved due to low response rates at 11.0% in the phase 1 study. The general decline in response rate is evident across all types of survey. The response rate of the telephone survey in the U.S. exceeded 56.1% in 2002, 42.4% in 2008 [33] and 18.0% in data reported in Germany in 2018 [34]. The survey responder in that study were compared on a wide range of social, economic and lifestyle measures from national database that include Statistics Korean and results were not significantly different [7]. Fifth, drug-induced nausea is one of the most common causes of nausea, however, there is no data on the use of drugs provoking nausea or the use of anti-emetic drugs in the phase 2 study [35]. In the present study, the presence of gastrointestinal damage caused by non-steroidal anti-inflammatory drugs or aspirin, a common cause of nausea caused by drugs, was excluded by the use of upper endoscopy, and the drug-induced nausea is most likely to develop acutely.

Sixth, gastroparesis is very rare disease diagnosed with gastric emptying test, but it has not been conducted to exclude it among the subjects with chronic nausea. However, the prevalence of gastroparesis per 100,000 persons is extremely low as 9.6 (95% CI, 1.8–17.4) for men and 37.8 (95% CI, 23.3–52.4) for women in the population based-study [36]. Based on this prevalence, there is a possibility that some 2 to 3 participants with gastroparesis of 5096 subjects are included. It is difficult to identify diabetic gastroparesis, therefore, we excluded uncontrolled diabetic patients who can affect gastrointestinal motor functions in phase 2.

In conclusions, the present study defined the clinically significant nausea that lowers HRQoL as having ‘more than one day of nausea per week. About 90% of clinically significant nausea was not accompanied with vomiting. The significant organic diseases cause chronic uninvestigated nausea were reflux esophagitis, duodenal ulcer and hyperthyroidism. After excluding the organic nausea, chronic unexplained nausea is rare at 0.6% (95% CI 0.4–0.8%), however, it is expected to cause the disease burden with common chronic comorbidities and poor HRQoL.

## Ethics statement

The authors confirm that the ethical policies of the journal, as noted on the journal’s author guidelines page, have been adhered to and the appropriate ethical review committee approval has been received. The study conformed to the US Federal Policy for the Protection of Human Subjects.

## Supporting information

S1 Table Baseline characteristics of uninvestigated nausea by Rome IV criteria in health check-up group; Phase 2 study. (DOCX)

S2 Table Possible organic causes of uninvestigated nausea by Rome IV criteria. (DOCX)

S3 Table Multivariate analysis for predictors of chronic unexplained nausea by Rome IV criteria. (DOCX)

S1 Data Data underlying the results and conclusions presented in the manuscript. (XLSX)
