# Supplementary material for: Chronic unexplained nausea in adults: Prevalence, impact on quality of life, and underlying organic diseases in a cohort of 5096 subjects comprehensively investigated
Source: PLoS One. 2019 Dec 19;14(12):e0225364. doi: 10.1371/journal.pone.0225364 (PMC6922349; doi:10.1371/journal.pone.0225364)
Supplement: S1 Table — (DOCX) [file pone.0225364.s001.docx]

|  | Controls  (n=4632) | Acute uninvestigated nausea (n=373) | Chronic uninvestigated nausea (n=91) | P value |
| --- | --- | --- | --- | --- |
| Age | 47.8 ± 9.9 | 45.7 ± 43.2 | 43.2 ± 11.1 | < 0.01 |
| < 40 years (%) | 900 (19.4) | 107 (29.7) | 30 (33.0) | < 0.01 |
| Female sex (%) | 2207 (47.6) | 203 (54.4) | 44 (48.4) | 0.04 |
| BMI (kg/m^2^) | 24.3 ± 25.0 | 23.2 ± 3.2 | 23.3 ± 3.2 | 0.66 |
| Obesity (≥ 25 kg/m^2^) (%) | 1728 (37.3) | 130 (34.9) | 28 (30.8) | 0.29 |
| Hb (g/dL) | 14.3 ± 1.6 | 14.2 ± 1.6 | 14.5 ± 1.7 | 0.37 |
| Albumin (g/dL) | 4.1 ± 0.3 | 4.1 ± 0.2 | 4.1 ± 0.3 | 0.23 |
| Total cholesterol (mg/dL) | 197.6 ± 34.2 | 199.4 ± 36.7 | 197.7 ± 35.7 | 0.63 |
| HDL-cholesterol (mg/dL) | 55.7 ± 13.2 | 57.3 ± 15.1 | 56.5 ± 12.2 | 0.06 |
| Triglyceride (mg/dL) | 118.6 ± 80.3 | 121.6 ± 106.6 | 124.2 ± 95.4 | 0.65 |
| r-GT (mg/dL) | 34.2 ± 38.7 | 37.9 ± 50.9 | 46.7 ± 55.6 | < 0.01 |
| ^†^ESR (mm/h) | 10.3 ± 8.1 | 10.3 ± 8.3 | 10.7 ± 9.4 | 0.33 |
| HbA1c (%) | 5.6 ± 0.7 | 5.5 ± 0.7 | 5.5 ± 0.6 | 0.11 |
| Alcohol (n=4986) (%) | 996 (22.0) | 115 (31.9) | 38 (41.8) | < 0.01 |
| Current smoker (n=4884) (%) | 799 (18.0) | 78 (21.9) | 31 (34.4) | < 0.01 |
| Diabetes mellitus (%) | 251 (5.4) | 15 (4.0) | 2 (2.2) | 0.21 |
| Hypertension (%) | 605 (13.1) | 49 (13.1) | 8 (8.8) | 0.49 |
| Asthma (%) | 203 (4.4) | 49 (13.1) | 14 (15.4) | < 0.01 |
| CVA (%) | 29 (0.6) | 0 (0.0) | 1 (1.1) | 0.47 |
| Coronary artery disease (%) | 64 (1.4) | 8 (2.1) | 1 (1.1) | 0.59 |
| Somatization | 13.1 ± 17.2 | 27.5 ± 29.5 | 38.4 ± 19.5 | < 0.01 |

**S1 Table. Baseline characteristics of uninvestigated nausea by Rome IV criteria in health check-up group; Phase 2 study**
