# Supplementary material for: Chronic unexplained nausea in adults: Prevalence, impact on quality of life, and underlying organic diseases in a cohort of 5096 subjects comprehensively investigated
Source: PLoS One. 2019 Dec 19;14(12):e0225364. doi: 10.1371/journal.pone.0225364 (PMC6922349; doi:10.1371/journal.pone.0225364)
Supplement: S2 Table — (DOCX) [file pone.0225364.s002.docx]

**S2 Table. Possible organic causes of uninvestigated nausea by Rome IV criteria**

| Number (%) | Controls  (n=4632) | Acute uninvestigated nausea (n=373) | Chronic uninvestigated nausea (n=91) | P value |
| --- | --- | --- | --- | --- |
| Organic disease | 649 (14.0) | 74 (19.8) | 17 (18.7) | < 0.01 |
| Reflux esophagitis | 284 (6.1) | 34 (9.1) | 8 (9.9) | 0.03 |
| Esophageal cancer | 1 (<0.01) | 0 (0.0) | 0 (0.0) | 0.95 |
| Gastric ulcer | 145 (3.1) | 11 (2.9) | 2 (2.2) | 0.87 |
| Duodenal ulcer | 71 (1.5) | 2 (0.5) | 3(3.3) | 0.09 |
| Stomach cancer | 7 (0.2) | 1 (0.3) | 0 (0.0) | 0.80 |
| Chronic liver disease | 175 (3.8) | 7 (1.9) | 3 (3.3) | 0.17 |
| Uncontrolled DM (n=4272) | 251 (5.4) | 15 (4.0) | 2 (2.2) | 0.21 |
| GB stone | 50 (1.1) | 4 (1.1) | 1 (1.1) | 1.00 |
| GB wall thickening | 18 (0.4) | 1 (0.3) | 0 (0.0) | 0.79 |
| Renal insufficiency | 1 (<0.01) | 0 (0.0) | 0 (0.0) | 0.95 |
| Renal stone | 100 (2.2) | 10 (2.7) | 3 (3.3) | 0.62 |
| Bradycardia | 3 (0.1) | 0 (0.0) | 0 (0.0) | 0.86 |
| Gastrectomy | 10 (0.2) | 1 (0.3) | 1(1.1) | 0.23 |
| Cholecystectomy | 29 (0.6) | 5 (1.3) | 0 (0.0) | 0.19 |
| Hyperthyroidism | 46 (1.0) | 5 (1.3) | 2 (2.2) | 0.45 |
| Hypothyroidism |  |  |  | 0.60 |

All Data are not mutually exclusive; Gastric and duodenal ulcer was included as having active or healing stage of ulcer; Uncontrolled DM, HbA1c >7; Renal insufficiency, CRF stage 3 or more.
