# Supplementary material for: Chronic unexplained nausea in adults: Prevalence, impact on quality of life, and underlying organic diseases in a cohort of 5096 subjects comprehensively investigated
Source: PLoS One. 2019 Dec 19;14(12):e0225364. doi: 10.1371/journal.pone.0225364 (PMC6922349; doi:10.1371/journal.pone.0225364)
Supplement: S3 Table — (DOCX) [file pone.0225364.s003.docx]

**S3 Table. Multivariate analysis for predictors of chronic unexplained nausea by Rome IV criteria**

|  | Univariate analysis | | | Multivariate analysis: model 1 | | | Multivariate analysis: model 2 | | |
| --- | --- | --- | --- | --- | --- | --- | --- | --- | --- |
| **N (%)** | OR | 95% CI | *p* value | OR | 95% CI | *p* value | OR | 95% CI | *p* value |
| Young age  <40 years | 3.01 | 1.47 – 3.73 | <0.01 | 2.07 | 1.28 – 3.33 | <0.01 | 2.38 | 1.37 – 5.28 | 0.02 |
| Female gender | 1.16 | 0.73 – 1.83 | 0.54 | 2.32 | 1.14 – 4.22 | 0.02 | 1.20 | 0.58 – 1.53 | 0.09 |
| Current smoking | 2.13 | 1.28 – 3.53 | <0.01 | 1.72 | 1.00 - 2.95 | 0.05 | 1.57 | 0.90 – 2.74 | 0.12 |
| Chronic alcohol use | 2.71 | 1.31 - 5.60 | 0.02 | 1.91 | 1.14 – 3.19 | 0.01 | 1.82 | 1.07 – 3.08 | 0.03 |
| Somatization | 8.31 | 4.00 – 17.24 | <0.01 |  |  |  | 6.53 | 3.99 – 10.7 | <0.01 |
| Asthma | 5.41 | 2.18 - 13.38 | <0.01 |  |  |  | 2.11 | 1.05 – 4.24 | 0.04 |

OR, odd ratio; CI, confidence interval; Alcohol use was defined as more than 61 g/week for males and 41 g/week for females.
